# Nurturing a positive research culture within your laboratory

**DOI:** 10.12688/wellcomeopenres.22492.1

**Published:** 2024-06-25

**Authors:** Adrian Liston, Denise C. Fitzgerald

**Affiliations:** 1 Department of Pathology, University of Cambridge, Cambridge, England, CB2 1QP, UK; 2 School of Medicine, Dentistry and Biomedical Sciences, Queen's University Belfast, Belfast, Northern Ireland, BT9 7BL, UK

**Keywords:** research, academia, research culture

## Abstract

As a Principal Investigator leading a research team, creating a positive research culture for your team members is one of the best long-term investments you can make, for your research programme, for the sector and for society. A positive research environment is one where team members are empowered, recognised, have a clear career developmental pathway, can contribute to impactful and reproducible research and, ideally, propagate these effects. While these virtues can arise organically from a team built around kindness and integrity, they are also values that should be deliberately embedded within your lab. Here we provide advice on how to create a culture of integrity and a culture of belonging for your team members. We focus on thoughtful consideration of your key lab values, and the use of structure, language and your personal actions to make these values explicit. A holistic approach to integrating positive culture throughout every facet of your research team creates a system that can be self-sustaining in scientific integrity and more resilient to negative challenges. Starting on the pathway to self-improvement as a manager, recognising that this requires often uncomfortable self-reflection, provides both personal and professional reward.

## Plain language summary

Running a research team is not simply about producing important research findings. It is important for the team leader to also focus on creating an environment that is a positive experience for the team members and a place for them to build further careers from. Focus on the culture of the team feeds back into the quality of the research, aiding impactful, reproducible and ethical research. Part of building a positive research environment comes from your role as a leader, and interacting with you team with kindness and integrity. However you can aid this process by engineering the structure of your team and through thoughtful consideration of your language and actions. In this article we discuss several tactics to research team leadership that may help team leaders create a positive environment for their team members.

## Disclaimer

The views expressed in this article are those of the authors. Publication in Wellcome Open Research does not imply endorsement by Wellcome.

Becoming a principal investigator (PI) and building a research team is an exciting and thrilling opportunity. For many it is the first chance to strike out independently, and test the ideas and skills that we have spent years developing. The job is both professionally and personally rewarding, with enormous opportunity for growth. It is also a time of great unknowns – stepping into a position long anticipated, but incompletely understood. There are often high expectations, from others and yourself, and a very real ticking clock. With a new environment to adapt to, and a new role to explore, the intangible “culture” of the team can often take a back seat to just getting experiments to work and delivering in the other aspects of your new role. Creating a positive research culture for your team members is, however, one of the best long-term investments you can make. A culture that is pleasant to work in, encourages and empowers individuals, and which works with cooperation and integrity, is not only the ethically right ambition, but also one that has the greatest long-term productivity. For those looking to start up their own research team, the earlier the culture is set, the more resilient it becomes to challenges, so we recommend early and recurring consideration of positive research culture practices as a core priority. For established PIs, while the best time to plant a tree is many years ago; the second best time is today. It is never too late to start positive research culture practices, or enhance your existing practice with new ideas. If your group’s culture is less than optimal, you can start to change it today. If upon reading this you, like the authors, uncomfortably recognise missed opportunities for you and your team, you are ideally placed to effect positive change.

## What is a positive research environment?

Every aspect of your research team structure, practice and interaction influences the research environment. A positive research environment is one where team members are empowered, recognised, have a clear career developmental pathway, and can contribute to impactful and reproducible research. As a PI, your role is, at its heart, managerial, and you have the core responsibility over the research environment you oversee.

Empowerment is the ability of each person to make impactful contributions to, and decisions about the laboratory / research team, including the space to make mistakes. Empowered decisions should be accompanied by group discussions, personal advice and consultation, to help identify the best decision. When all team members have a clear demarcation of the domain that they lead on, they feel trusted and respected, and take the responsibility more seriously. Standing by your team members’ decisions includes supporting decisions that did not pay off, and helping them to find mitigation strategies for those mistakes.

Recognition of contributions is a key aspect to any positive research environment. This will obviously include recognition on publications, through authorship and formal acknowledgements, proportionate to their contribution and not their position. More often forgotten are the recognition of non-publication roles and of developmental progress. Non-publication roles include formal recognition of supervisory and administrative roles; in particular, any jobs that are undertaken by the team member should be accompanied by assigning the title to accompany the job (e.g. advocating for the team member to have the official title of assistant supervisor, health and safety officer, collaborative/team lead etc). Developmental progress recognition involves providing new opportunities for team members commensurate with their experience (reviewing papers, writing commentaries, presenting at conferences, managing collaborations), and promoting their progress to the institutional and external community (nomination for awards, promoting achievements, writing reference letters).

Having a clear career development pathway is essential to reduce the anxiety and insecurity that underlies many of the most toxic aspects of a research career. This does not impart responsibility for the team member’s career on you; indeed, an empowered team member should feel responsible for their own career. However, it does require transparency and a long-term perspective on your behalf, so the team member understands the duration and nature of commitment they have from you and the type and extent of support you can offer. This should be flexible and compassionate in the face of unexpected change. Most importantly, the time spent in your laboratory should not simply focus on accomplishing the goals you set out for the period, but should leave the team member with an expanded range of experience, skills and opportunities for the future.

Finally, the research environment should enable a contribution to impactful and reproducible research. This goes well-beyond running experiments and publishing. It encompasses protocol development, access to resources and the ability to work on important questions. It involves training and support in experimental design, statistics, reproducibility, collaborative work, skills in mentoring and supervision and ethical and open science. An expanded view of impact needs to include resource/data/methodology-sharing, knowledge exchange, public engagement, training others, influencing clinical practice, technology transfer and conference presentation.

In an ideal situation, the prioritisation of a positive research culture will already be present within your host organisation, and will be promoted and reinforced through positive institutional policies and practices. In other cases, a laboratory will be embedded within an organisation with less ideal practices, and in some cases the PI will even need to take on an insulation role to prevent external negative research cultures becoming established within their team. We have separately discussed approaches to enhance the positive research culture at the organisational level ^1^; here we discuss key strategies for integrating positive practices at the laboratory level.

## Setting standards and expectations

The culture of a laboratory can be rapidly absorbed by new members through interactions with the team. However, these values can be taken up faster and more consistently by explicitly communicating your values and expectations to new team members. 

There are multiple ways to communicate expectations to your team members. These can include informal education from team members, formal one-on-one induction sessions, semi-regular team presentations, jointly filling out expectations surveys ^2^, or a written document. We would advocate for a written document to be included in this process, even if another mode is the primary used, due to the benefits of allowing later referral. In many labs this written communication takes the form of a “lab policies”, “lab manual” or “lab guide” document, which lay out the logistics and resources of working in the lab – how ordering systems work, how booking systems operate, travel policies, lab meeting details, communication norms, lab duties, safety documents, access to internal and external facilities, and so forth ( Box 1). A well-crafted guide can also communicate values, both explicitly and implicitly. A starting point for these guides is often the document from a previous host. Consider also asking a lab that you admire the research culture of, for a copy of their guide. Open access templates are also available ^3^.

**Box 1. Logistics reflect values**

Even the nuts-and-bolts logistics of lab operations reflect values. For example:

    •   Policies on data storage that include shared protocols and shared servers hosting data communally are more consistent with the values of transparency and teamwork.

    •   Policies on communication platforms like Slack or Basecamp that allow all team members to view and comment updates will encourage broader participation.

    •   Travel policies that guarantee conferences to all staff members and are linked to time rather than publications remove pressures to achieve the “right” outcome on experiments.

    •   Communication lists like phone numbers can explicitly state the situations they should be used (e.g. a freezer failure, or animal welfare issue) to signal work-life separation.

Consider looking at your lab logistics with fresh eyes – what values do they signal?

The lab guide is a great place to embed expectations of research culture and integrity. Beginning the guide with a description of the lab mission is an effective way to set the stage. A good mission statement will not simply emphasize the scientific goals, but also the methods (robust and reproducible science) and the non-scientific goals (e.g. training and mentorship, impacting the ecosystem through your alumni, broadening participation in STEM, educating the public). Ask yourself why you are running a lab, and then put that answer down on paper for the lab mission statement. It is also worth returning to that mission statement annually, and asking yourself whether your actions in the year were consistent with your ideals. As your group evolves it is useful to seek feedback from your team on your mission statement. Is it clear? Does it represent to them why and how the team functions?

Beyond the mission statement, the lab guide can cover your approach to research culture. You can include your expectations of working in the lab, your policies towards data-sharing, what team members can expect to receive in terms of mentorship and professional development, your ideals for inter-personnel behaviour, ethics in science (in particular when working with patient samples/data and animals), principles for experimental design and analysis, sources for professional and personal help. In each case, taking a holistic approach to integrating integrity and positive culture into your approach builds a synergistic culture. For example, “expectations” that focus on output create a pressurised system, while expectations that focus on approach can reinforce values of good experimental design and integrity. A guide should also encompass yourself as part of the team – behaviour and ethics that you expect your team to follow should be binding on yourself. Indeed, your own behaviour will impact culture stronger than your written word – a policy that encourages respectful interactions needs to be modelled by your interactions with the team. Adding your personal perspective, as to *why* key policies are in place, provides team members with an insight into your values and strengthens the impact of those policies. 

While the “lab guide” can be a single document, we have found it helpful to have three versions. A “lab overview” document, that gives insights into potential lab members as to the culture and expectations, can be given to any potential recruit, to give them the information they need to decide whether the lab is a good fit for them. A “welcome” document can be used for onboarding, when someone accepts the job, with just the key information for weeks leading up to joining and the first days after joining. The full “lab guide” then covers all the detailed information for current lab members.

The lab guide is not the only way to explicitly communicate your values. Every interaction with your team communicates your values implicitly; every interaction has the potential to include explicit explanation. For example, a staff member requesting a day off, and getting a quick “no problem” is an implicit communication of the value of flexibility. A more detailed response to the same request (especially from a new recruit) could explicitly describe the value: “Of course! I trust you to manage your own time, you never need to request permission from me for time off or holidays, please just organise your work around the time off”. Likewise, your willingness to share your own failings, such as an unsuccessful grant or being wrong about an experimental result, communicate the value that failure is normal and shared, and thus make it easier for your team members to share failed or expectation-violating experiments. Team-wide communications (whether by email or during a lab meeting) in particular set standards and expectations, and the tone of this communication can be as important as the content. For example, a negative email complaining about the tissue culture hoods being left messy again could instead strengthen positive values if it is framed as an affirmation of the value of shared space and a reminder that our daily actions can make everyone’s work more pleasant. It is often worth considering incorporating the relevant communications into the lab guide, so that new lab members have access to the information.

## Building a culture of integrity

**Figure 1.**
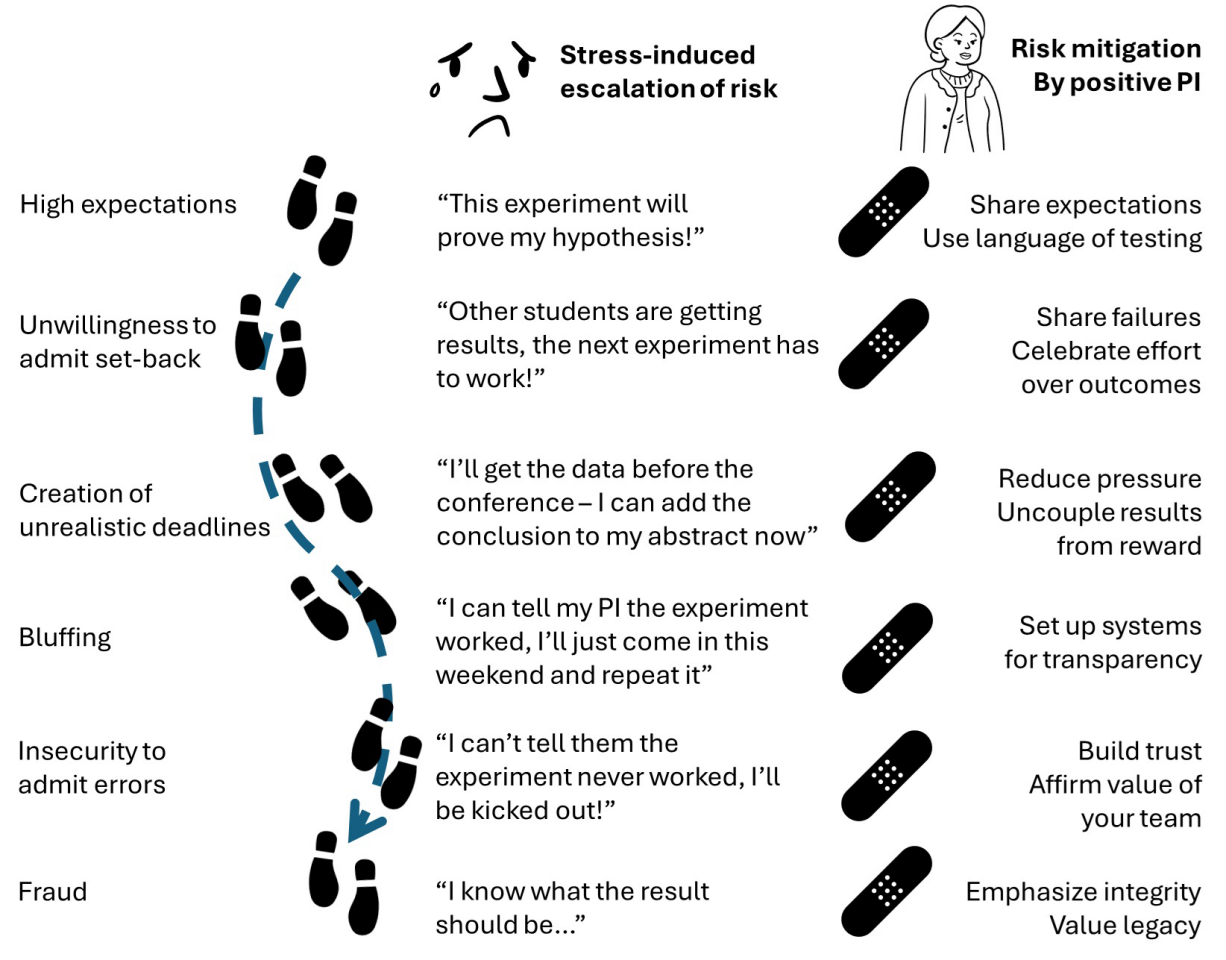
The pathway from high expectations to fraud. Scientific misconduct can arise even in people with good intentions. (Left) A combination of stress such as overly high expectations, unrealistic pressures and insecurities can make people feel like fraud is the only answer. (Centre) An example of individual small steps leading to data fabrications. (Right) Examples of how a positive research culture could have prevented or mitigated the conditions that led to fraud at each stage.

Scientific fraud can seem like an alien idea to many of us. When the scientific career is a calling, based on finding new truths, the concept of contaminating the scientific literature with deceptions is a perversion. While no system can prevent all cheating, the best defence against scientific fraud is an understanding of the pressures and small steps that can lead a well-intentioned researcher towards it ( Figure 1). In many ways, an undergraduate degree in science does not prepare a student for the repeated failure of experimental science in practice. Students move from an arena of studying, where effort is reasonably proportional to outcome, to the arena of research, where failed experiments (often involving huge effort) and unknown outcomes are the norm. They move from a system of being rewarded for demonstrating their factual knowledge (getting the right answer) to a system of determining what isn’t known, and working through ways to answer questions that don’t yet have known answers. If the definitions of success are not changed and the expectations are not moderated, early career researchers develop intense internal pressure. External pressure, from perceived success and expectations in the environment, especially if coming from the PI, can be unbearable. Team members may receive signals that prioritise and reward success (getting the “right” result) over method (well-designed experiment).

Building a culture of integrity within your laboratory is not simply a matter of endorsing and even modelling behaviours of integrity. A resilient culture of integrity needs to be vigilant for the pressures that create incentives for cheating, and to actively counter those pressures. These pressures can be structural, such as deadlines, expectations on the outcomes of experiments, harsh responses to mistakes, environments of data secrecy, and inter- and intra-lab competition. More innocently, but still potentially harmful, the pressures can simply be linguistic – consider the impact of “we need to do this experiment to prove that our cells promote inflammation” versus “we need to do this experiment to test whether our cells promote inflammation”, or “we need to boost that n to make the result significant” versus “we need to replicate the experiment to see if the result is reproducible”. Getting your language right every time is unrealistic, but the more you practice use of integrity-focused language the more natural it becomes. You can start by making a habit of reviewing the text that you write for team members. Does the lab email celebrating a paper acceptance focus on the success of “getting past the reviewers” or on the long-term commitment to quality research that will impact the field? Do your replies to figures being sent to you focus on whether the result matches your expectations, or whether the design and data quality are high? In lab meetings make a point of being intrigued when the results conflict with your expectations. In planning sessions estimating time-lines, be explicit that you expect experimental failures and diversions. With new team members it can also help to be explicit about the role of integrity-promotion in the decisions you make, as you cannot assume that they have been trained with similar priorities in past positions. An environment where the path-of-least-resistance is honesty and transparency is one where mistakes made are admitted and learned from; an environment which celebrates method over outcome is one which will encourage reproducibility.

## Going beyond the “Golden Rule”

**Figure 2.**
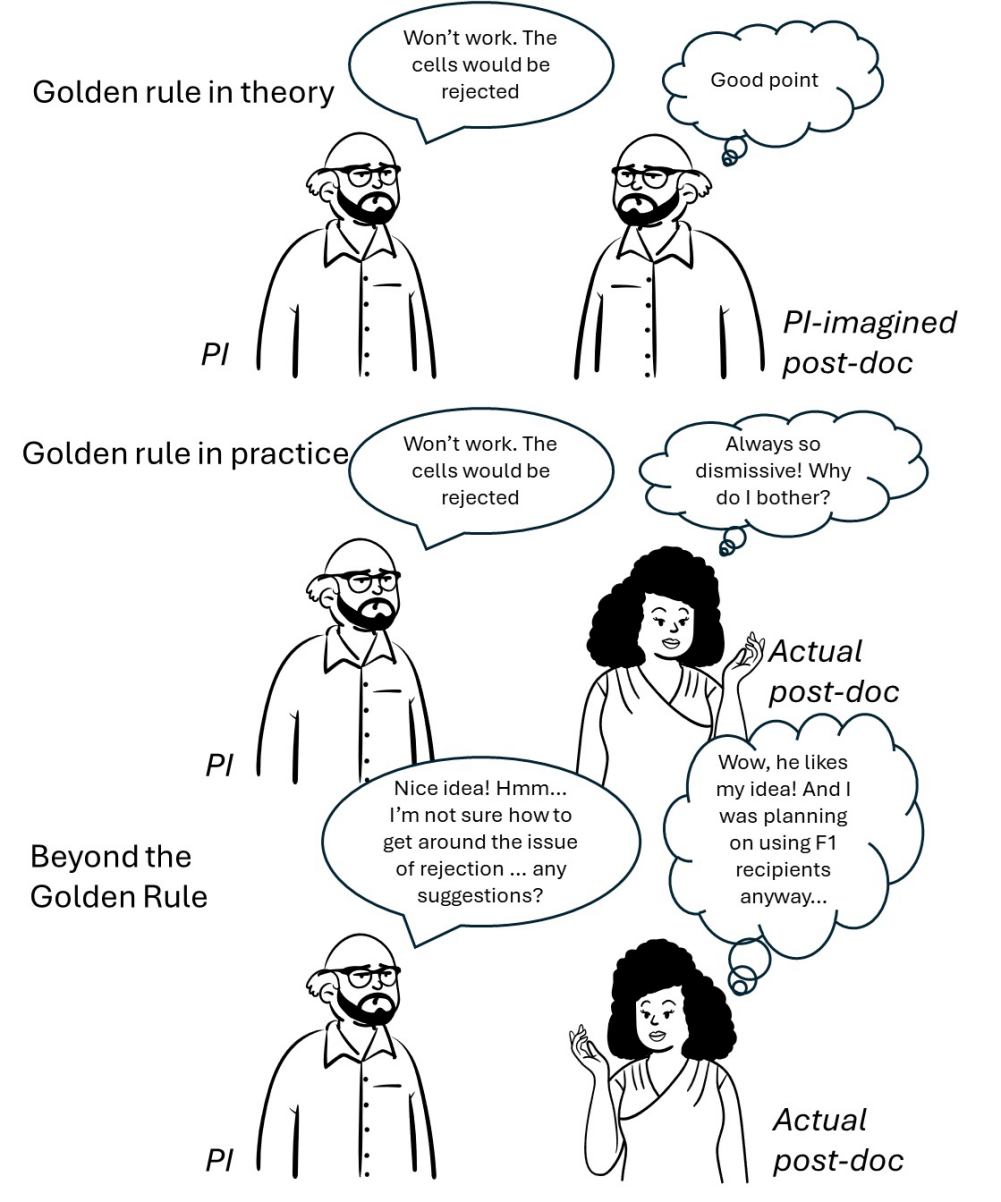
Beyond the Golden Rule. Treating your team members as you would like to be treated is a good first step, but falls down when your expectations differ from your team members. Understanding your team members as individuals, who will respond differently to you, helps you tailor the signals of respect that you send to them. Talking to people through their own language of respect, understanding generational change and background differences, is more effective in building a relationship that enables communication.

The “Golden Rule”, treat everyone as you would like to be treated, is a positive first step as a PI. Under pressure and stress, people often forget to treat their team members with the courtesy that they expect in return. We would advocate, however, that creating an inclusive research culture means going beyond this Golden Rule, and instead treat everyone as *they* need to be treated to reach their growth zone. The distinction lies in a recognition that we are all different – we all come with different experiences, different cultural lenses, different expectations, different filters. Using the way that you would like to be treated as the baseline for respect makes the assumption that your team members are budding clones of yourself. A simplistic example of this phenomenon would be a PI who is immune to negative criticism, and appreciates the efficiencies of blunt feedback. Using the same language on other people can be dispiriting and shut-down communication ( Figure 2). In particular, a PI needs to be sensitive to the fact that everyone carries with them unique emotional “scar tissue”, where their interactions with you will be filtered through their past experiences. A new team member coming from a toxic lab where people were shouted at for mistakes will need gentle support to learn to become comfortable again to admit mistakes.

There are no short-cuts to going beyond the Golden Rule. It involves caring about every team member as an individual, and taking the time to listen to their story. Lab social activities, team-building exercises, mentoring sessions – these are all opportunities for you to learn about your team members. If you genuinely understand and care about your team members as individuals, and talk their language of respect to them, it will profoundly alter your lab dynamic for the better.

## Building a sense of belonging and the importance of ritual

A sense of belonging builds a group of individuals into a team. The lab should feel like a safe space, with shared ownership, and team members should feel included as part of a greater endeavour. While the extent to which team members feel friendship to each other will naturally vary, and as a PI a degree of professional distance is necessary, everyone should enjoy being in the workplace. The small touches can make a huge difference here – a nook to have a shared coffee in, people sending back post-cards while on a conference or returning with an exotic treat, potted plants and message boards, an end-of-year dinner and going to seminars together. Entering a lab to a smile and looking forward to catching up with other team members sets the tone for the entire day.

A sense of belonging is not necessarily an easy thing to build, as it depends so much on the personalities and interactions of other lab members. It can be enabled by structure (allowing personalised space rather than hot-desking, providing break facilities that promote interaction), be modelled by your own behaviour (take the time to greet and farewell everyone, ask about people’s day and actually listen), and be kick-started through lab events (dinners, team-building exercises, annual retreats, sporting events). New students, especially undergraduate students, may need to be explicitly welcomed into the team – the transition from student to team member is a profound one. Celebrations of milestones is an important part of being in a team, however, remember to align your language with your values, as glorifying the result rather than the effort/approach can send the wrong message to your team. Remember that a diversity of approaches has the best chance of finding an avenue that every team member has something to look forward to. In particular, be wary of the use of alcohol-centred events. While serving alcohol has the advantage of flagging the shift to informality, high consumption can create long-term problems, and even limited consumption narrows the inclusiveness of the event. PIs can learn from younger generations, who often have a more moderate relationship with alcohol, about creating a relaxed informal environment with low, or no alcohol.

On the importance of ritual, most of us start our careers in science wanting to make a difference. Asking around your team for their motivations, it will not be uncommon to hear of a loved one getting ill, a desire to improve society, or just curiosity to find out how the universe ticks. The practice of science operates on a profoundly different time-line to this ambition. This discrepancy is especially acute for early career researchers taking their first steps from science in the media or in textbooks to science as an experimental endeavour. As a PI, your experience provides a longer perspective; you have seen projects start as ideas, germinate slowly, and hit rough patch after rough patch before reaching fruition. Even the success of whole projects is often just one stage in their lifecycle, as it can take many years or decades for the true value of a discovery to unwind.

**Figure 3.**
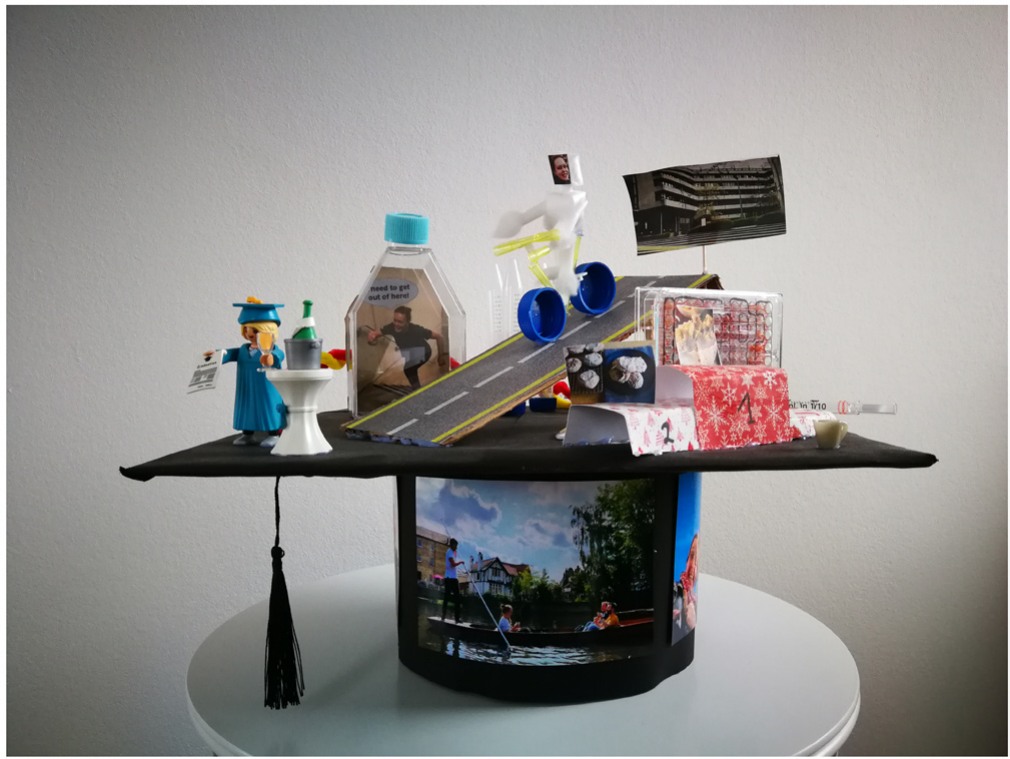
The German doctoral graduation hat tradition. Our version of the German graduation hat tradition involves the current PhD currents getting together in advance of the graduation to craft a feature mortarboard. The hat includes small items, often made from lab disposables, and photo prints, reflecting poignant moments from the PhD. The hat is then presented to the graduating student at the lab celebration, and is often a highlight of the event! Example hat from Dr Julika Neumann.

**Figure 4.**
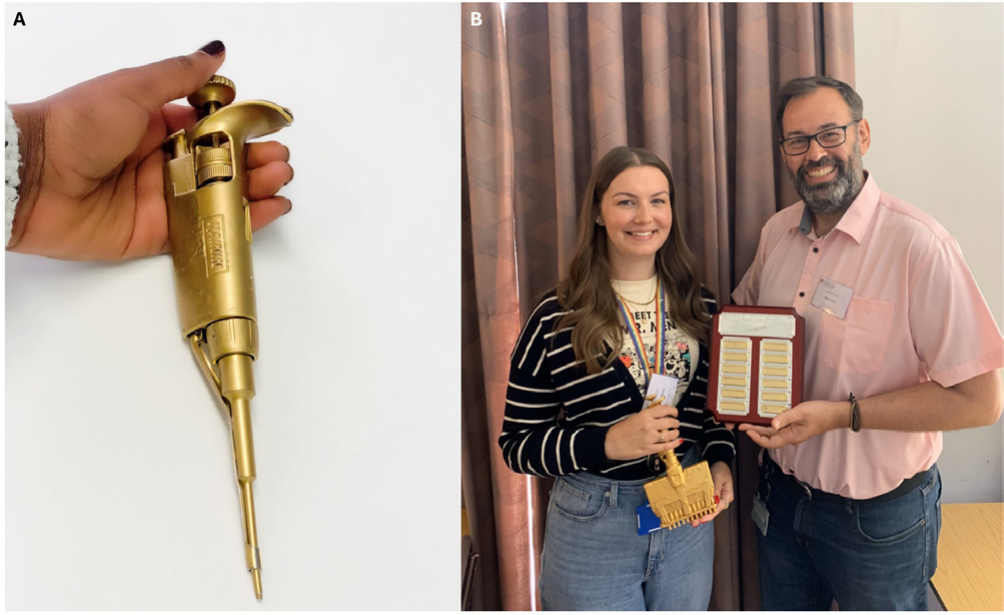
The Golden Pipette. A long-running tradition in our lab is the awarding of the Golden Pipette. **A**) A broken pipette, painted gold, was deliberately given a mythical backstory and awarded (along with prize money) for an elegant experiment presented at our annual lab retreat. Presented by winner Ntombizodwa Makuyana. **B**) The winner of the re-vamped golden pipette, here Amy Dashwood, is now also engraved on a prominent plaque, and the award has been re-shaped towards team contributions, reflecting a value choice.

**Figure 5.**
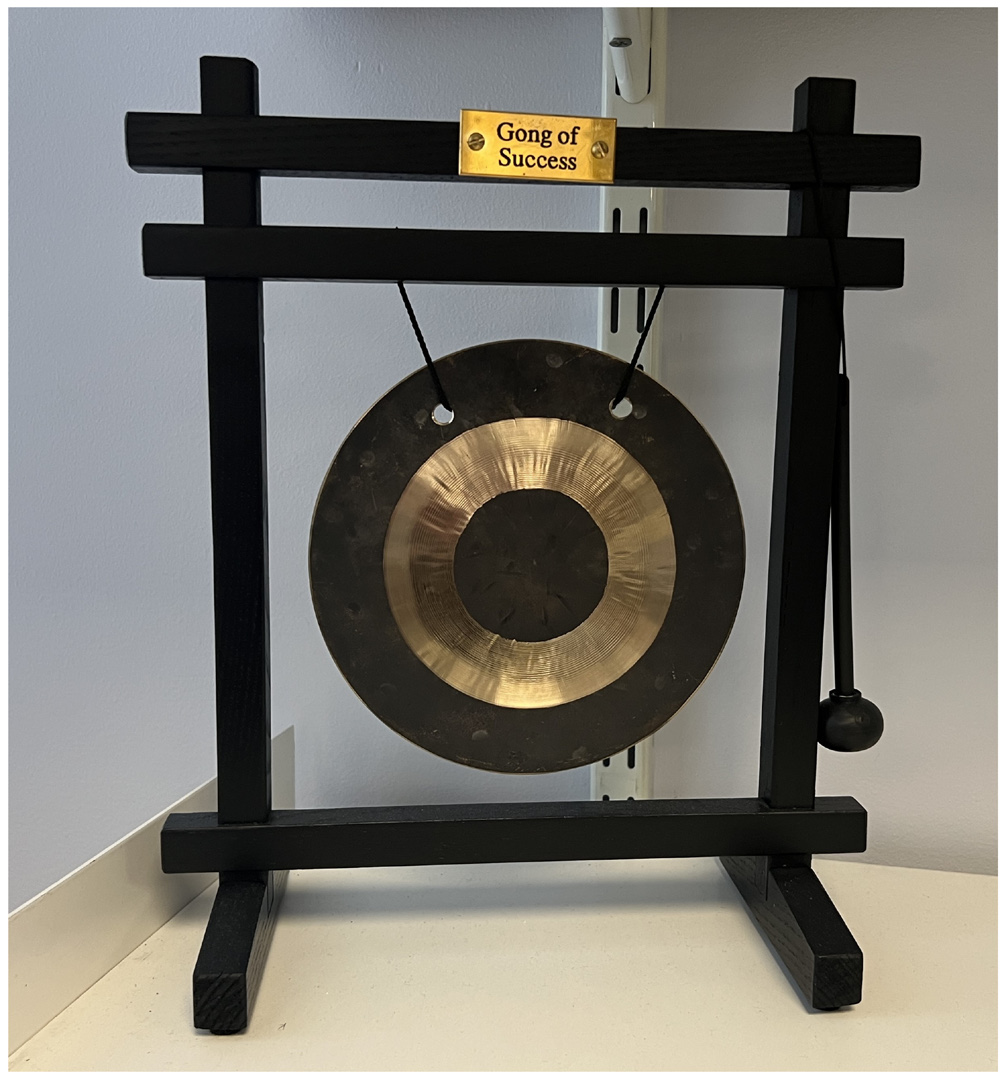
The Gong of Success. A recent tradition we are setting up in the lab is the Gong of Success, to be rung on achieving any success, large or small. While not yet well-established in the lab, the gong ringing certainly attracts attention and provides an opportunity to share that your PCR finally worked, you finished a draft of your methods, or your abstract got selected for a conference.

A valuable message you can impart upon your lab is the aggregate impact of many small steps. Seeing oneself as part of a relay-race, taking over the baton from earlier lab members and passing it on to future lab members, is a useful perspective to counter the incremental nature of small projects. Such an approach prioritises values such as robustness of results, consistency of protocols, reliability of conclusions and quality of recordkeeping, while de-emphasizing individual glory and splashy conclusions. These values can, and should, all be directly imparted during training and lab interactions, however they can also be indirectly built on through lab “rituals” or “traditions”. These traditions can be simple, such as everyone going out for coffee together on Tuesday afternoons, or complex, such as the German hat tradition ( Figure 3), the Golden Pipette ( Figure 4) or the Gong of Success ( Figure 5). As with celebrations, the choice of what to uphold as a tradition is a clear measure of your values – consider measures that are linked to integrity and process over result. While new traditions initially feel forced, the rapid turn-over of lab members soon means the tradition has been going longer than most of the lab. Such traditions, by their nature, create the sense of being a part of something larger than yourself. Use of alumni networks (e.g., a LinkedIn group is easy to set up and add members to) and story-telling of past challenges and successes, can impart a feeling that current members will be long remembered and are essential for future successes. Bringing up the profound impact that current team members will have on future team members and projects is a great way to demonstrate that you value them and their contribution now and into the future.

## Feedback and career development

Team members need feedback to get a good understanding of their current performance and future trajectory. Scientific training is critique based: we seek to disprove our hypotheses, knock down models that we have generated, look for flaws in experiments, identify weaknesses in grants, search for the problems in papers. In many ways, the average PI is over-trained in criticism, and under-trained in compliments – identifying the strength of an experimental design, appreciating an elegant control or low technical variation, acknowledging the novelty of an idea, or praising a well-written paper. When this over-training spills into management practices, the effect can be dispiriting, especially for those new to research. Take the time to provide praise to your team members, frequently and in person and in writing. This can be even more impactful when in public such as at a joint lab meeting or retreat, where your supportive culture can propagate to other teams. Often enough you will need to also impart criticism (see below), but always taken the time and opportunity to acknowledge the positive. Simple gestures like complimenting someone for putting forward their ideas, praising someone for their team contribution, giving appreciation for the quality of experimental design or execution, are all appreciated by your team members. The nature of the positive praise is also a route by which you impart your values – by praising robust and reproducible design, collaborative work, data-sharing, and so forth, you directly encourage more of this behaviour in your lab. Be mindful of what may be a major challenge or accomplishment for one person may not be for another. For example, presenting research in front of others may be a huge challenge for some. Overcoming this challenge is not only a major achievement of growth for them but also a valuable opportunity for you to provide positive feedback and recognition of their efforts. This requires knowing what your individual team members find challenging and supporting them to grow through these challenges.

An essential part of feedback involves a discussion of career development. For those that don’t yet know what they want to do next, or long-term, it is important to sign-post career-exploration strategies and re-visit on a regular basis. Frequently, those that don’t know what they want next also struggle with the discomfort of trying to find out and prefer to bury their heads in their projects. Having career development as a set agenda item at regular meetings provides a prompt both for them to devote time to the topic, and for you to discuss it routinely. For those that have a clear goal, it is useful for team members to understand how far they are on track for their career goals. For you to give this feedback you need to understand what the career goals for your team member are and what limitations may apply to their choices e.g. income requirement, geographic location, mode of working. We suggest that these discussions should take place at least once a year (but ideally more), with more frequent discussions at key inflection points. We advocate for a structured progression of expanding opportunities, where regular career discussions progress from identifying potential career trajectories, to exploring and laying down career markers in those trajectories, to building the required profile to enact a post-lab career decision ^4^. Individual Development Plans can be useful tools to frame this process, albeit with the acknowledgement that career paths rarely develop exactly as anticipated! Even for staff members with indefinite positions in your laboratory (e.g. staff scientists and technicians), career development discussions are essential, and career development opportunities to upskill and diversify experiences and networks/contact should be provided.

Several common themes often emerge from career development meetings. At initial stages many team members will not have a defined career goal. Mentoring for such team members should include reassurance that this is normal, and a discussion that career decisions are not final. It is enough simply to think of several potential careers that could be of interest in a next step. This gives enough information to later provide opportunities for your team member to “taste” those career pathways, and to solidify around 3-4 potential career development routes. Other team members may be overly fixed on a single career goal, often that of becoming a PI. Mentoring for these team members should include attempts to broaden horizons. Ask what aspects of being a PI appeal to the team member, and suggest other career pathways that provide similar opportunities, broadening to 3-4 potential career development routes. One of the most useful things a PI can do is to explain how much the role of PI can differ from a PhD or Postdoc and that this usually diverges even further with time, career progression and group size. Having identified potential career pathways, provide opportunities for your team member to test their interest, lay down markers of experience and build credibility in the field. For those interested in an academic career, apprenticeship-style approaches in your daily practice are invaluable. All team members should also be considering non-academic careers, which may mean drawing on external resources (career office, Postdoctoral Development Centre, your personal network, your alumni network) to provide those opportunities. Often matching your team member with additional mentors with the appropriate wisdom will prove valuable. Similarly, helping them to identify mentors they click with themselves, broadens their support base and their network. While we can intend to be an impartial mentor for our team members, our priorities as PI can sometimes conflict with team member career goals and this is where external mentors become particularly helpful. Finally, the wind-down period of your team member’s time in your lab should be planned with a compatible time-scale to build applications, apply for positions/grants and transition to the next stage.

Good career mentoring, and positive post-lab career outcomes, do not only benefit the exiting team member. Your alumni are the key ambassadors for your lab; word of the culture and opportunities in your lab travel with them. Seeing team members get mentoring and move to exciting career opportunities is also a comfort and major stress-relief for your more junior team members, who see the opportunities that will be provided to them. The caveat to this is the inadvertent pressure some may feel to match the achievements of their predecessors; here it is important that your team members identify what their specific personal career goals are, and acknowledge when then progress towards these goals. Finally, the value of “reverse mentoring” should not be understated – the process of learning the perspectives and challenges of your team members provides you with a continually updated understanding of the research career, and makes you a more empathetic and more effective manager.

## Giving growth-directed feedback

One of the biggest challenges of having your own team as a PI is learning how to communicate with them effectively about with the areas that you wish your team member to grow and improve in. This can be difficult on both the “when” and the “how”. For this growth-directed feedback (and we would encourage PIs to think in this term, rather than “negative feedback”) to be effective, the aim has to be about correcting the issue and avoiding it reoccurring in the future. Both you and your team member need to be in the right mind frame for an open discussion, and the approach and language used needs to be conducive to positive outcomes.

On the “when”, it is important to give space on both sides to see how a situation pans out – self-correction on their behalf or acceptance of an alternative style on your behalf can both be positive outcomes if given time. On the other hand, even mild annoyances can damage a relationship if they are not brought up openly and discussed at an early enough stage. An honest introspection is also important in deciding when to bring up an issue – has the issue arisen because of your actions? Are you raising the issue for the sake of the team member, or because of its impact on you or others in the team? Either way, the issue can be raised, but having an honest self-assessment of the context in advance will aid the conversation. For example, if a team member is late for an agreed deadline, it is worth considering questions such as whether the team member fully briefed on what needed to be done, whether the team member had enough experience to do the project within the timeframe, whether you have sent mixed messages to the team on the flexibility of timeframes, whether you clearly articulated priorities, whether the behaviour is actually detrimental or if it is just a “pet peeve” of yours ^5^, and so forth. It is also useful to determine if there may be extenuating circumstances that are not immediately obvious. As a general rule, the frequency of growth-directed feedback on minor points is often well accepted in the lab, as it can calm a worry that you hide major issues, as long as a) you are even more frequent on your praise-directed feedback, and b) you are receptive to receiving growth-directed feedback from your team members in return. As always, your own behaviour ends up being the most powerful signal of expectations. If there is a temptation to vent, you are not ready to give appropriate negative feedback. If you can’t get past your frustration, it can be useful to talk through options with a trusted colleague to decide on what approach may lead to best outcomes all round.

More important than the “when” is the “how”. Always remember the power discrepancy at play. As a supervisor / line-manager / employer, your words will be heard with a heavier weight and implication than you may mean. Imagine how you would feel if your Departmental Chair started this conversation with you. Plan ahead your first lines to get the conversation off to a good start. Discussions that start out with an accusation will usually put the team member on the defensive. For our example of a missed deadline, starting with “you missed the deadline” can trigger a fear response. Even when it is controlled it can cause an emotional shut-down, severe anxiety, counter-attacks, excuses or rationalisations. Starting on a related positive note will create a more conductive start: “thank you for taking this task on for me, I really appreciate it”. There are several different approaches that can be used, including the “compliment sandwich” (with praise-growth-praise feedback), admissions of self-responsibility (“I apologise for giving you this responsibility too early, I misjudged your readiness and I am sorry if this put you under stress”) and the use of “I language” rather than “you language” ( Box 2). Regardless of the strategy you take, it is important to ensure that the point is internalised and understood while taking the time to ensure that your staff member leaves feeling valued. This can take more time than you may expect – only start a growth-directed feedback session if you have at least twice as much time available as you expect the session to take, as it can be damaging to your relationship to cut off such sessions abruptly.

**Box 2. Using “I language”**

In the example issue of a missed deadline, the topic can be brought up with “I language” or “you language” ^6^.

"You language" would be saying "you missed this deadline". The problem with this is that it immediately puts the person on the defensive, triggering responses such as such as “you gave me too much to do at once”.

"I language" reframes the debate. "I felt disrespected that you missed this deadline and gave it to me late. I specifically asked for it on Thursday, because I had reserved Friday morning to work on it, and I’ll be busy next week. Because I got it a day late, I had to skip out on going to the park with my son on Saturday. I know that this doesn’t sound like a big deal, but when I manage a team of 15, people only need to do this twice a year to make me feel like I am constantly neglecting my family".

The advantage of "I language" is that it gets to the real problem – your emotion response to the issue. It puts the other person in a zone of empathy rather than in a zone of defence. They will feel less attacked, taken into your confidence, and will gain an insight into what is important to you. It may feel odd “justifying” your annoyance, but if the aim of the conversation is to reduce repeat behaviour, the “I language” approach is often more effective.

A small note on “where”. This should rarely be a difficult issue. Conducive discussions about growth-directed feedback should almost never be in a public space. If discussions start in the open lab and head in that direction, move them to a private space. One area where this can be tricky is in lab meetings, where critical evaluation of data can be perceived as, or in reality lead to, growth-directed feedback that would be more suitable for a private setting. Remember that conversations can always be stopped and brought up later, and you can course-correct in real-time: “let’s talk about the controls later, there is a lot of interesting work here, let’s see the next slide!”.

## Building trust as a continual process

A positive research culture should be resilient to set-backs; an atmosphere of mutual trust and respect is an atmosphere that forgives mistakes and allows second chances. It is important, however, that the positive research culture is continually strengthened, reinvigorated and propagated by the team as a whole. This will also involve periodic reinvention – expectations and standards evolve with time, and PIs need to reflect whether their ideals and strategies are still appropriate ( Box 3). For example, a lab policy or lab guide document will not only need continual updating in order to remain useful, but should on occasion be rewritten if the tone has become inconsistent with the desired messaging. New practices can be brought in to counter a potentially worrying trend, and old practices can be discontinued if the outcome is no longer positive.

**Box 3. Reflections on current practice**

**Step 1. Dedicate time to reflect on the current culture of the lab.** This can be done in bite-sized pieces concentrating on one aspect, e.g. your lab policy document or lab meeting structure. External collaborators can also be useful to feedback on culture, for example at annual retreats.

**Step 2. Identify cultural aspects that you would like to see improved.** For example, you may hope for more interaction during lab meetings.

**Step 3. Consider any structural barriers that may be causing the problem, and remove them.** For example, if lab meetings are set too late in the day, people may be holding back on discussion to finish at a more reasonable time. Remember to consider structures from the point of view of your team members, not yourself.

**Step 4. Consider any personal changes you can make to promote the desired culture.** Positive actions from you may be needed to flag the expectation. For example, compliment the ideas brought up by lab members publicly in lab meetings, and follow up and thank them for sharing their ideas. Alternatively, you may be taking negative actions that inhibit the culture, such as interrupting or dismissing comments, which you can stop.

**Step 4. Consider any cultural changes that would promote improvements, and implement trials.** It may be appropriate to bring lab members in on potential solutions; team members will have novel insights into potential solutions. The act of asking (and then listening without dismissing!) is also a statement of value which can create changes by itself. A one-on-one question on the lines of “I’m always impressed by your scientific insights. I’m wondering how I could encourage you to share them with the rest of the lab at lab meetings?” can be far more conducive than sending out a bulk email telling people that they need to be more interactive.

**Step 5. Monitor your behaviour for consistency with new ambitions, and course correct.** As PI, your behaviour signals more strongly than your words, so you need to take greater responsibility for the positive change you want to enact. Monitor how often you are remembering to use your positive influence (e.g. compliment feedback) and how often you are backtracking on negative influences. Consider setting yourself reminders, e.g. a written note on your lab meeting notebook saying “Wait before jumping in!” or a reoccurring calendar reminder to thank interactive team members.

An important consideration in reinventing your positive research culture is the evolving relationship between you and your team as your career progresses. At early stages of our careers we face different challenges. Being fresh to the role allows for a greater understanding of the generational context of your trainees, and is often associated with closer working together at the bench and a more flexible approach to trialling management innovations. On the other hand, it is also accompanied by less experience, a less-developed network to draw upon, and, especially for women, a perceived “authority gap” which can be difficult to overcome without institutional support. At later stages in your career, the generational and professional gap between you and team members will widen, providing new challenges. For example, your memories of the financial context of being a post-doc become increasingly irrelevant the further removed your post-doc time becomes. Even if you take care to grow your understanding of the shifting challenges of new team members, limiting the knowledge gap, the perception of the gap will grow in the eyes of your team members. An example of this is that the perceived gravity of your feedback can strengthen with your seniority, and so you may need to soften your language over time to achieve the same impact. This effect can take PIs by surprise, as their actions are consistent, but the perception of those actions shifts.

Finally, it is worth reflecting that “the team” is a collection of individuals, acting at different time-scales. A PI who has continually invested in positive research culture will build up a trusting relationship with the team, and the team as a whole will pick up the values of the PI. However an academic lab has continual turnover, and that relationship needs to be rebuilt with every individual. Senior PIs, often with increased external draws upon their time, may not realise that they have under-invested in the relationship building needed for each new generation of team members. A misevaluation of the degree of mutual trust with the team can be problematic: it is too easy, at times, to rely on banked trust with the team (e.g. a long-term reputation for being understanding of the difficulties of science) to buffer mistakes (such as getting frustrated at a poorly designed or executed experiment). When mistakes are made with recent team members the buffer is weak, and the budding relationship can be damaged if you do not recognise and rectify your mistake. In particular, if your lab enlarges or if additional duties reduce the time you spend with individual lab members, remember to make an active effort to reaffirm your positive research culture values with all of your team members. A few simple chats over coffee can go a long way to building rapport and breaking down perceived barriers. 

## Conclusions

An overarching theme of these guidelines is understanding and kindness. Just as you should approach short-comings from your team with understanding and kindness, so it is essential to apply these virtues to yourself. As we have found during the process of writing this manuscript, articulating your philosophy on research culture is one of the most effective ways to develop your approaches to research culture, however it can also be profoundly uncomfortable. The process forces one to identify past mistakes, acknowledge our agency in them, and consider how situations could have been averted or solved. Self-reflection, while confronting, is the process through which we grow. Opportunities for such self-reflection are many – applications for funding and promotion should include time to reflect on past and future practice. Writing reference letters for team members provides an opportunity to consider the positive aspects they brought to the team. Discussion with colleagues about their practices can provide beneficial insights into our own, especially when we reach out to a broad and diverse set of colleagues. Our team members, if in an atmosphere of trust, can provide the most potent advice: a 360 feedback session on research culture can identify places you have failed to live up to your values, or miscommunicated those values. Lab management/leadership courses are available, and can serve a valuable role in providing both the time and space to reflect on your practices.

No matter how good our intentions or thorough our preparations, at some point every PI will fail to live up to their expectations, every PI will misjudge the impact of their words, every PI will let their team down – we are human. If, on reading this article, you identify problematic aspects to your past or current practice, you are not alone. We are advocating for these positive research culture practices not from a position of success, but from being on the path of continued improvement. In the process of writing we have identified further opportunities for improvement in our own practice. It can be easy to dwell on self-criticism, however it is more beneficial to take the opportunity to support your growth through your discomfort on your personal and professional development. Hopefully you can take your insights as opportunities to model the behaviour you want to see from team members – acknowledge the failure, apologise, commit to improvement, and follow-through on that commitment. These are the actions of a secure leader, one who is not afraid of being seen to mess up. Fortunately, if you have cultivated a positive research environment in your laboratory, your team should respond with understanding and kindness in return.

## Data Availability

No data are associated with this article.
