# Supplementary material for: Nurturing a positive research culture within your laboratory
Source: Wellcome Open Res. 2024 Oct 15;9:341. Originally published 2024 Jun 25. [Version 2] doi: 10.12688/wellcomeopenres.22492.2 (PMC11443193; doi:10.12688/wellcomeopenres.22492.2)
Supplement: Peer review history

# Reviewer report: Reviewer response for version 2

**DOI:** 10.21956/wellcomeopenres.25589.r107033

**Published:** 2024-11-14

**Authors:** Alberto Antonietti

**Affiliations:** 1 Department of Electronics, Information and Bioengineering Politecnico di Milano, Milano, Italy

The Open Letter presents the challenges that PIs face in their daily lives when managing a research team, with a thorough discussion on how to establish a positive and welcoming environment and to create the foundations for the flourishing of younger members (e.g., thesis students, PhD students, and postdocs.) The authors present many different suggestions and various methods for doing this, presenting established good practices that worked, at least from the authors' experiences.

 As a young PI myself, I found the text very well-written and informative. I enjoyed reading it and I believe it reached its goal of stimulating self-reflection on my current practices.

 I have just some minor suggestions:

- In the introduction, it could be worthwhile to mention that most of the suggestions are applicable in a "standard-sized" lab (e.g., less than 10 members: 1 PI, 1-2 postdocs, 3-4 PhD students). I believe the suggestions are not feasible with larger research teams.
- One message that I think it should be passed is that we, as PI, have to be aware that the "it has been always done like this in the past" way of thinking is very dangerous. Sometimes we have the tendency to think that it is OK to perpetuate old (bad) practices believing they were needed to arrive at the position where we are now and that these experiences "forged" us. Discussing this issue could be a useful addition to the paper. 
- A couple of years ago we wrote a consensus paper on a similar topic, from the perspective of early career researchers: "Recommendations for empowering early career researchers to improve research culture and practice" Kent BA *et al.* (2022 ^1^)

I believe it is an interesting further reading that could be added to the references.

Does the article adequately reference differing views and opinions?

Yes

Are all factual statements correct, and are statements and arguments made adequately supported by citations?

Yes

Is the Open Letter written in accessible language?

Yes

Where applicable, are recommendations and next steps explained clearly for others to follow?

Yes

Is the rationale for the Open Letter provided in sufficient detail?

Yes

Reviewer Expertise:

Bioengineering, Neuroengineering, Open Science

I confirm that I have read this submission and believe that I have an appropriate level of expertise to confirm that it is of an acceptable scientific standard, however I have significant reservations, as outlined above.

## References

[1] : Recommendations for empowering early career researchers to improve research culture and practice. *PLoS Biol* .2022;20(7) : 10.1371/journal.pbio.3001680 e3001680 10.1371/journal.pbio.3001680 35797414 PMC9295962

# Reviewer report: Reviewer response for version 2

**DOI:** 10.21956/wellcomeopenres.25589.r108448

**Published:** 2024-11-11

**Authors:** Daniel Hammett

**Affiliations:** 1 The University of Sheffield, Sheffield, England, UK

The article sets out a 'call to arms' for the development of more supporting and positive (research) cultures within scientific lab teams. The work provides an overview of a range of ways in which team cultures can be enhanced and supported, ranging from discussion of the individual practice and language choices of the PI through to collective behaviours and (invented) lab traditions to support and celebrate achievement. Many of these suggestions may feel obvious to some, but unfortunately not to everyone - and a clear and concise outline of the importance of good practice is a valuable addition to work in this field. 

 While I find myself in broad agreement with the sentiments and approaches set out here - and I read this from a social science background where research teams are rarely 'lab-based' in the style assumed in this paper - a few areas for reflection come to mind. 

 Firstly, I wondered to what extent the issue of/styles of practice that encourage negative, or positive research cultures are informed by previous personal experience. This emerges implicitly in the paper, but a little more direct commentary might be considered - something leading to my second point. 

 My own mentor's approach was the mantra of 'pay it forwards' - their intention (and expectation - which was made explicit) being that by their supporting my development and positive practice, I was morally obligated to then 'pay forwards' by providing mentorship and support to those I managed/supervised in future. This idea(l) is clearly implicit across this paper, but I wonder if the authors might like to draw out this longer-term legacy aspect further? That by delivering on the idea(l)s set out in the paper, we might see a broader trend of culture change. 

 One challenge I was a little surprised not to see mentioned (and this might well be due to the difference in disciplinary backgrounds at work) was the common (inevitable?) tension for supportive PI's between wanting their team to excel and develop their own careers by taking up further opportunities when available, and the disruption this may then cause to the PI's team and project. This may be something less pronounced/obvious in larger teams but can be a concern for smaller (social science) teams. 

 One final thought in relation to nurturing positive research cultures relates to ensuring an open and genuine commitment to EDI (Equality, Diversity and Inclusion) policies and practices - and ensuring a zero-tolerance approach to bullying, harassment, and the like - and how these needs to not only be modelled by the PI and other senior members of a team, but through clear and accessible reporting procedures. Linked to this, it was notable that sociability and team building was mentioned - with the move to reduce the centrality of alcohol to these activities noted: could further consideration be noted here relating to the timing and location of events (a particular consideration for carers and parents for instance).

 A short paper such as this cannot cover every eventuality or consideration, and some of my comments might be expecting a little too much given the space available to the authors. Overall, this is a really accessible and engaging paper - one that should become key reading for those setting up (or indeed, already leading) their own labs/teams.

Does the article adequately reference differing views and opinions?

Partly

Are all factual statements correct, and are statements and arguments made adequately supported by citations?

Yes

Is the Open Letter written in accessible language?

Yes

Where applicable, are recommendations and next steps explained clearly for others to follow?

Yes

Is the rationale for the Open Letter provided in sufficient detail?

Yes

Reviewer Expertise:

Political geography, research ethics, citizenship

I confirm that I have read this submission and believe that I have an appropriate level of expertise to confirm that it is of an acceptable scientific standard.

# Reviewer report: Reviewer response for version 2

**DOI:** 10.21956/wellcomeopenres.25589.r107029

**Published:** 2024-11-06

**Authors:** Thanikachalam Vedhathiri

**Affiliations:** 1 National Institute of Technical Teachers Training and Research, Chennai, India

1. Build a strong foundation for your leadership approach.
2. Leverage learning modalities to support the research associates.
3. Build excitement and grow your research program. 
4. Foster collaboration
5. Research culture campuses the behavior, values, expectations, attitudes, and norms of the research communities.
6. Mentor research associates to propose clear objectives.
7. Promote learning and innovation.
8. Guide to prepare outstanding reports and papers.
9. Include limitations, if there are any.
10. Validate the findings/ model/ concepts
11. Choose an appropriate research setup (Free from errors)
12. Check the literature and identify the gaps

Does the article adequately reference differing views and opinions?

Partly

Are all factual statements correct, and are statements and arguments made adequately supported by citations?

Yes

Is the Open Letter written in accessible language?

Yes

Where applicable, are recommendations and next steps explained clearly for others to follow?

Yes

Is the rationale for the Open Letter provided in sufficient detail?

Yes

Reviewer Expertise:

Engineering Education Research, Educational Management, Institutional Development, Leadership Development, Outcome-based and Flexible Curriculum, Diverse Global Faculty Development, Human Resources Development, Interdisciplinary Research.

I confirm that I have read this submission and believe that I have an appropriate level of expertise to confirm that it is of an acceptable scientific standard.

# Reviewer report: Reviewer response for version 2

**DOI:** 10.21956/wellcomeopenres.25589.r105612

**Published:** 2024-11-04

**Authors:** James P Takona

**Affiliations:** 1 Coppin State University, Baltimore, Maryland, USA

We have carefully reviewed the manuscript on creating a positive research culture within scientific teams. The manuscript is commendable in its depth and approach, offering relevant insights into fostering empowerment, recognition, and career development. Its focus on integrity and kindness as core values is both timely and constructive, potentially serving as a valuable resource for new and experienced principal investigators alike.  Very timely and commendable work!

 However, we noted several areas where author's manuscript could benefit from further refinement:

1. **Practicality and Specificity:** While the manuscript offers a solid conceptual framework, it often leans on generalizations that may feel abstract to readers. We recommend incorporating more specific, actionable examples or step-by-step strategies, particularly in sections on feedback and career development. This would make the guidance more directly applicable.
2. **Challenges and Realistic Applications:** Currently, the manuscript lacks acknowledgment of potential obstacles a PI may face, such as institutional limitations or resource constraints. Including a section such that it addresses "Common Challenges and Solutions" would offer readers a more balanced and realistic view of implementing these practices.
3. **Diversity and Inclusivity:** Although there is a general call for understanding and kindness, the manuscript could benefit from a deeper exploration of inclusivity and diversity within research teams. That might simply be the dedicated section on fostering an inclusive environment, with strategies for accommodating diverse perspectives, would strengthen the manuscript's relevance.
4. **Readability and Structure**: Certain sections are clearly dense, which may hinder readability. We suggest breaking up these sections with subheadings, lists, or visual aids such as flowcharts or summary tables to make the document more navigable and engaging.
5. **Audience Inclusivity:** The manuscript often assumes that PIs have a high degree of autonomy in decision-making. Revising some of the language to acknowledge diverse experiences, including those in more restrictive or resource-limited settings, would help broaden its appeal.

These enhancements would increase the manuscript's clarity and practicality, providing a robust resource for PIs across various research environments. We believe these changes would make the manuscript even more impactful and valuable to the target audience.

 We have carefully reviewed the manuscript on creating a positive research culture within scientific teams. The manuscript is commendable in its depth and approach, offering relevant insights into fostering empowerment, recognition, and career development. Its focus on integrity and kindness as core values is both timely and constructive, potentially serving as a valuable resource for new and experienced principal investigators alike.

 However, we also noted several areas where the manuscript could benefit from further refinement:

1. **Practicality and Specificity:** While the manuscript offers a solid conceptual framework, it often leans on generalizations that may feel abstract to readers. We recommend incorporating more specific, actionable examples or step-by-step strategies, particularly in sections on feedback and career development. This would make the guidance more directly applicable.
2. **Challenges and Realistic Applications:** Currently, the manuscript lacks acknowledgment of potential obstacles a PI may face, such as institutional limitations or resource constraints. Including a section that addresses "Common Challenges and Solutions" would offer readers a more balanced and realistic view of implementing these practices.
3. **Diversity and Inclusivity:** Although there is a general call for understanding and kindness, the manuscript could benefit from a deeper exploration of inclusivity and diversity within research teams. A dedicated section on fostering an inclusive environment, with strategies for accommodating diverse perspectives, would strengthen the manuscript's relevance.
4. **Readability and Structure**: Certain sections are dense, which may hinder readability. We suggest breaking up these sections with subheadings, lists, or visual aids such as flowcharts or summary tables to make the document more navigable and engaging.
5. **Audience Inclusivity:** The manuscript often assumes that PIs have a high degree of autonomy in decision-making. Revising some of the language to acknowledge diverse experiences, including those in more restrictive or resource-limited settings, would help broaden its appeal.

These enhancements would increase the manuscript's clarity and practicality, providing a robust resource for PIs across various research environments. We believe these changes would make the manuscript even more impactful and valuable to the target audience.

Does the article adequately reference differing views and opinions?

Partly

Are all factual statements correct, and are statements and arguments made adequately supported by citations?

Yes

Is the Open Letter written in accessible language?

Yes

Where applicable, are recommendations and next steps explained clearly for others to follow?

Partly

Is the rationale for the Open Letter provided in sufficient detail?

Partly

Reviewer Expertise:

While I'm fluent in research including its mechanics, in this particular study, I am able to assess only some areas of the study.

I confirm that I have read this submission and believe that I have an appropriate level of expertise to confirm that it is of an acceptable scientific standard, however I have significant reservations, as outlined above.

# Reviewer report: Reviewer response for version 1

**DOI:** 10.21956/wellcomeopenres.24783.r99199

**Published:** 2024-09-30

**Authors:** Paul Ndebele

**Affiliations:** 1 George Washington University, Washington, DC, USA

This article describes various strategies that principal investigators can apply in improving research cultures in their labs. 

 The strategies are practical in nature and easy to follow.  From reading the paper, the reader is left with a feeling that the authors are assuming that other individuals in the lab are always ready to cooperate with the PIs as they implement the various strategies. 

 Authors did not adequately address the issues of resistance that the PIs may face. Authors also did not adequately address the role of the institutional environment and what strategies the PI may implement to insulate the lab from external influences.

Does the article adequately reference differing views and opinions?

Partly

Are all factual statements correct, and are statements and arguments made adequately supported by citations?

Yes

Is the Open Letter written in accessible language?

Yes

Where applicable, are recommendations and next steps explained clearly for others to follow?

Yes

Is the rationale for the Open Letter provided in sufficient detail?

Yes

Reviewer Expertise:

Bioethics, Public Health

I confirm that I have read this submission and believe that I have an appropriate level of expertise to confirm that it is of an acceptable scientific standard.

# Response

**Published:** 2024-10-04

**Authors:** Adrian Liston

**Affiliations:** Department of Pathology, University of Cambridge, Cambridge, England, UK

Thank you. We have added the following paragraph in response to the issue of resistance: 

 1. A possible challenge in implementing best practices may be the willingness of all team members to engage with these activities. Two mitigation strategies here include ensuring open communication between current and candidate new team members during the recruitment process and co-development of new strategies with genuine, active input from the team, for the good of the team. Open communication will help both candidates and team leaders to understand each others expectations of how a team should function, and co-production of new strategies will support buy-in by team members." With regards to the institutional resistance, this is a critical point, but here the topic is far too large to address. Instead we have referred to our recent paper on this specific topic:

 "We have separately discussed approaches to enhance the positive research culture at the organizational level [1]."

# Reviewer report: Reviewer response for version 1

**DOI:** 10.21956/wellcomeopenres.24783.r96939

**Published:** 2024-09-17

**Authors:** Lisa M. Rasmussen

**Affiliations:** 1 Department of Philosophy, The University of North Carolina, Charlotte, USA

This article offers a useful narrative regarding steps for nurturing a positive review culture within a lab. It is clear, well-written, nuanced, and should be quite helpful, particularly for new faculty setting up their own research groups. I look forward to using it myself as a recommended reading for mentor training at my institution.

 The article starts by acknowledging how important a positive culture is for fostering not only more success and happiness for research and researchers, but also for more reliable and rigorous research. As the authors note, it is not enough to be kind oneself, values "that should be deliberately embedded" in a lab.  What follows is a very helpful (and thorough) set of recommendations and examples about how to do that.

 The only suggestion I want to offer is that these recommendations would be helpful beyond labs and into the broader realm of research groups. I'm sure the authors don't actually mean to restrict their recommendations to "the lab," but it might help to have a simple statement towards the beginning noting that while the authors focus on their familiar area of lab culture, what they have to say would be helpful to most research groups, however they are constituted.

 There are a few typos remaining in the article; I didn't note them all, but for example, on p. 9, line 9 in the left column, I think it should read "but always take the time" rather than "but always taken the time." On the same page, right column, about 9 lines from the bottom, it should say "when they progress" rather "when then progress".

 I think the article is clear and useful, no other comments!

Does the article adequately reference differing views and opinions?

Yes

Are all factual statements correct, and are statements and arguments made adequately supported by citations?

Yes

Is the Open Letter written in accessible language?

Yes

Where applicable, are recommendations and next steps explained clearly for others to follow?

Yes

Is the rationale for the Open Letter provided in sufficient detail?

Yes

Reviewer Expertise:

Research ethics and integrity; education in the responsible conduce of research; mentorship; authorship

I confirm that I have read this submission and believe that I have an appropriate level of expertise to confirm that it is of an acceptable scientific standard.

# Response

**Published:** 2024-10-04

**Authors:** Adrian Liston

**Affiliations:** Department of Pathology, University of Cambridge, Cambridge, England, UK

Thank you. We have corrected the typos and added the following pointer as suggested:

 "While we have written this from the perspective of lab-based research teams, many of the principles can be applied to other types of research teams"
